# Pharmacological and analytical aspects of alkannin/shikonin and their derivatives: An update from 2008 to 2022

**DOI:** 10.1016/j.chmed.2022.08.001

**Published:** 2022-09-20

**Authors:** Kirandeep Kaur, Rashi Sharma, Atamjit Singh, Shivani Attri, Saroj Arora, Sarabjit Kaur, Neena Bedi

**Affiliations:** a Department of Pharmaceutical Sciences, Guru Nanak Dev University, Amritsar 143005, India; b Department of Botanical and Environmental Sciences, Guru Nanak Dev University, Amritsar 143005, India

**Keywords:** alkannin, naphthoquinones, patents, shikonin

## Abstract

Alkannin/shikonin (A/S) and their derivatives are naturally occurring naphthoquinones majorly found in Boraginaceae family plants. They are integral constituents of traditional Chinese medicine Zicao (roots of *Lithospermum erythrorhizon*). In last two decades significant increase in pharmacological investigations on alkannin/shikonin and their derivatives has been reported that resulted in discovery of their novel mechanisms in various diseases and disorders. This review throws light on recently conducted pharmacological investigations on alkannin/shikonin and their derivatives and their outputs. Various analytical aspects are also discussed and brief summary of patent applications on inventions containing alkannin/shikonin and its derivatives is also provided.

## 1 Introduction

**Fig. 1:**
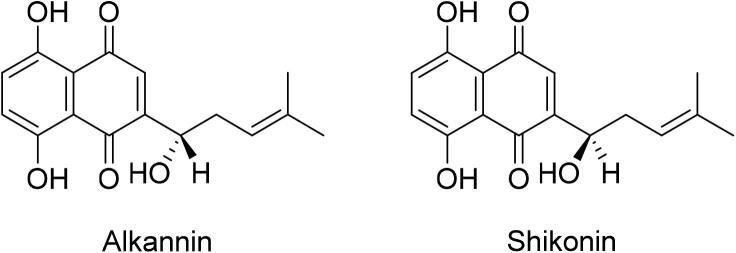
Chemical structures of alkannin and shikonin.

Alkannin and shikonin (A/S) are enantiomeric pair and naphthoquinone pigments (Boulos, Rahama, Hegazy, & Efferth, 2019) which are well known for their therapeutic, cosmetic and coloring applications **(**Fig. 1**).** Plants containing these bioactive pigments are traditionally used for curing various ailments since centuries. Alkannin was initially reported as a principle component of the root bark of with records of traditional utilization for 4th century BCE for various ailments, principally for ulcers (Papageorgiou et al., 1999, Weigle, 1974). On the other hand, Alkanna tinctoria Tausch. Plant of European Origin belonging to Boraginaceae family shikonin was isolated from the root bark of Chinese medicinal plant *Lithospermum erythrorhizon* Sieb. et Zucc (Boraginaceae) which is well known in China by various traditional names i.e. tzuts’ao, tzu-ken, hung-tzu ken, etc (Huu Tung, Du, Wang, Yuan, & Shoyama, 2013). It is an integral component of traditional Chinese medicine Zicao (roots of *L. erythrorhizon*) which has successful history in treatment of various inflammatory and infectious diseases (Andújar, Ríos, Giner, & Recio, 2013, Papageorgiou et al., 1999, Winter, 1984a, Winter, 1984b, Khan and Abourashed, 2010).

**Fig. 2:**
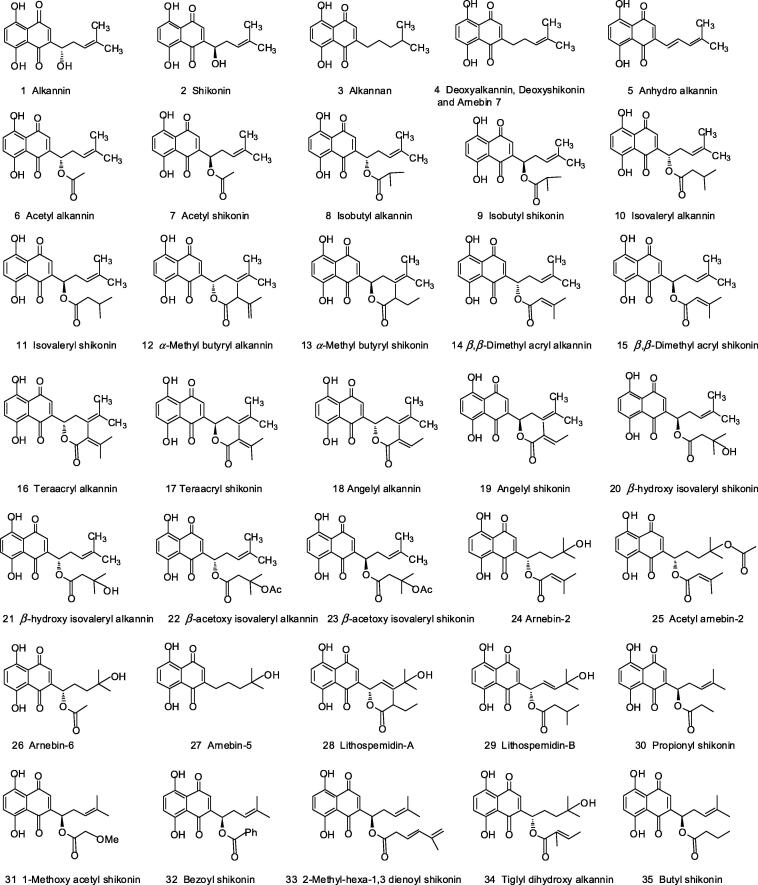
Structural representation of alkannin, shikonin and their derivatives.

Apart from these plants, a wide range of plants belonging to Boraginaceae family are known to possess these enantiomers and their derivatives. In fact acetylshikonin was first isolated from *L. erythrorhizon* by Kuroda and Wada in 1922, later shikonin and its other derivatives were also identified (Kuroda & Wada, 1936). It took almost 14 years to identify accurate structure of shikonin (reported as 5,8-dihydroxy-2-[(1*R*)-1-hydroxy-4-methyl-3-pentenyl]-1,4 naphthoquinone in 1936 by Brockmann); Subsequently, it’s another enantiomer alkannin was identified by the same group (Albreht et al., 2009, Brockmann, 1936). Approximately, 35 derivatives **(**Fig. 2**)** of alkannin and shikonin have been isolated from various plants of Boraginaceae family and extensively investigated for wide range of biological activities including wound healing, antimicrobial (Aburjai, Al-Janabi, Al-Mamoori, & Azzam, 2019), anti-acne (Fang & Shoukang, 1998), antiulcer (Singh & Sharma, 2012), anti-inflammatory (Lee et al., 2016), anticancer (Sun, Zhang, Liu, & Guan, 2019) activities, etc.

**Fig. 3:**
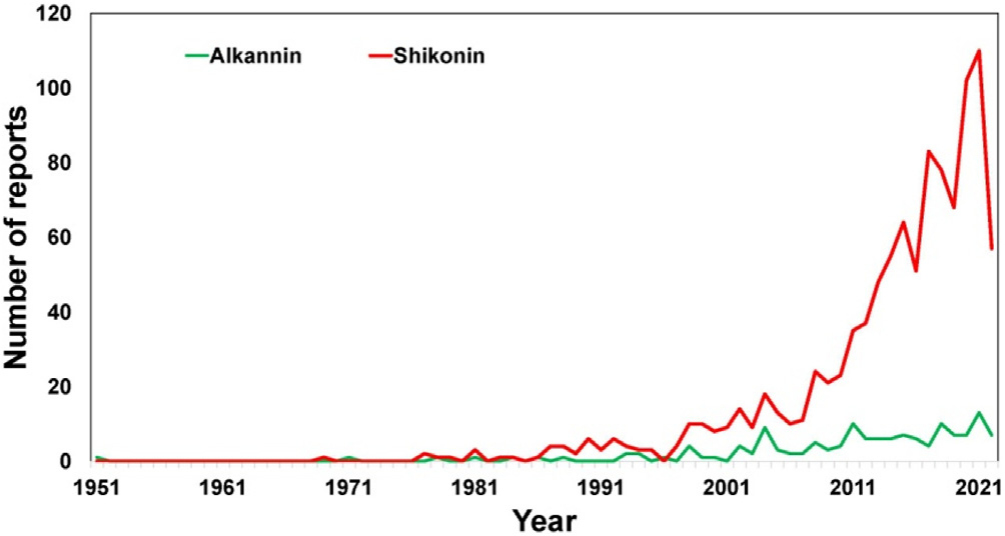
Trend analysis of reports indexed in “PubMed” database with keywords “Alkannin” and “Shikonin” from 1951 to 2021.

In the time frame of 1969 to 2021, a total of 634 full text reports are available in PubMed database and out of these, and 606 reports are published after 2000, showing the increased interest of research groups in A/S and their derivatives. Trend analysis suggests that researchers are more focused on shikonin than alkannin **(**Fig. 3**).** An exhaustive review of A/S and their derivatives was first published by Papageorgiou group in 1999 (Papageorgiou, Assimopoulou, Couladouros, Hepworth, & Nicolaou, 1999). A decade later, another update was published with prime focus on wound healing and associated bioactivities (Papageorgiou, Assimopoulou, & Ballis, 2008). In 2013, Andujar group published a compilation containing pharmacological investigations on A/S and their derivatives for the period of 2002 to 2013 (Andújar, Ríos, Giner, & Recio, 2013). Subsequently, various review reports were published by different research groups with a focus on either individual bioactivity or on individual derivative. A/S and their derivatives possess enantiomeric properties that make their analysis quite complex. Surface-enhanced Raman Spectroscopy (SERS) and chiral HPLC have been successfully utilized for differentiating A/S and their derivatives (Cañamares, et al., 2022; Azuma et al., 2016). Literature analysis suggests that after 2008 (Papageorgiou, Assimopoulou, & Ballis, 2008), any review update regarding analytical aspects of A/S and their derivatives is not available. Thus, there is a dire need of an updated compilation containing all pharmacological, analytical and miscellaneous investigations on A/S and their derivatives. Forecasting the marketable potential of A/S and their derivatives, wide range of patents have been filed by various research groups around the globe for various applications to safeguard their usage. This review is primarily focused on providing update on various investigations on A/S and their derivatives from year 2008 to 2021 along with thorough insight on the patent applications filed.

## 2 Pharmacological activities

### 2.1 Wound healing activity

Dried roots of *Arnebia guttata* Bung, *Arnebia euchroma* (Royle) Johnston, and *Lithospermum erythrorhizon* Sieb. Et Zucc loaded oil based ointment (Zicao) has been widely used for treatment of wounds (Chak, Hsiao, & Chen, 2013, Hsiao, Tsai, & Chak, 2012, Lu et al., 2008, Zeng & Zhu, 2014). The major active components of Zicao include shikonin and its derivatives such as deoxyshikonin, acetylshikonin and β,β’-dimethylacrylshikonin. Furthermore, to overcome the demerits of this oil based ointments such as discomfort, irritation and difficulty in cleaning, soluble water based topical preparation such as Zicao-HP-β-CD complex was formulated using 2-hydroxypropyl-β-cyclodextrin to form water-soluble complex which resulted in its enhanced bioavailability and stability. The active ingredients of Zicao enhance collagen synthesis in granuloma tissues and promote inactivation of tumor necrosis factor-α gene expression (Chen, Yu, Hsu, Tsai, & Tsai, 2018). On the other hand, Jawoongo, a Korean traditional medicine has been found highly effective in removing necrotic tissue caused by burn wounds. Jawoongo consists of *Lithospermi Radix, Angelicae Gigantis Radix, Ronicerae Flos, Glycyrrhizae Radix, Coptidis Rhizome* and *Scutellariae Radix*. The major active ingredient is *Lithospermi Radix* which mainly comprises of deoxyshikonin. It significantly increases the phosphorylation of p38 and ERK1/2 in a concentration dependent manner. Additionally, it activates Mitogen-activated protein kinase (MAPK) signaling which promotes cellular migration and angiogenesis. It was observed that deoxyshikonin induced migration and proliferation in HaCaT cells mediated through activation of p38 and ERK respectively. Thus, the study demonstrated that deoxyshikonin possesses strong ability for proliferation, migration and tube formation of HaCaT and HUVEC cells, which in turn promotes angiogenesis (Kim, Lee, & Yook, 2013, Park et al., 2017).

**Fig. 4:**
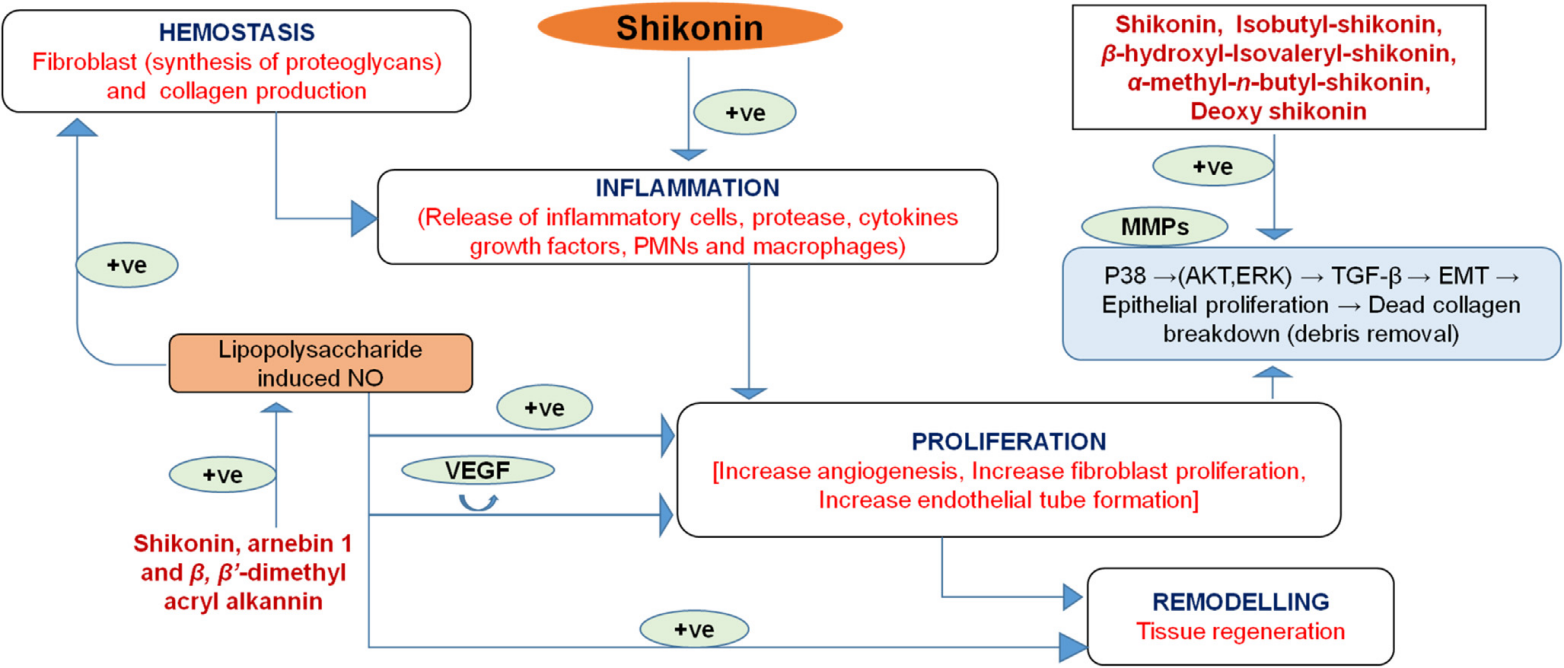
Impact of shikonin and derivatives on different phases of wound healing.

Recently, an increased attention is focused on the herbal medicines attributing to their quality, safety and efficacy. Since ancient times, people have used plant based preparations to promote wound healing process (Fronza, Heinzmann, Hamburger, Laufer, & Merfort, 2009). Various plants especially belonging to Boraginaceae family have been reported to possess excellent therapeutic potential in wound management. The main active metabolites of this family are naphthoquinones which possess anti-inflammatory, anti-microbial, anti-oxidant activities contributing to wound healing (Lee et al., 2016). Meanwhile, additional studies demonstrated that therapeutic benefits of roots of Boraginaceae family plants are wider than its aerial parts. The most active components found in roots are shikonin, alkannin, deoxyshikonin and acetylshikonin. Traditionally, the root extract of *Onosma dichroantha* Boiss. has been used in Iran for healing burn wounds. Furthermore, the cyclohexane fraction has been found to be most potent inhibitor of lipopolysaccharide induced nitrogen oxide production which accelerates fibroblast proliferation, tissue regeneration and angiogenesis. Active components present in the cyclohexane fraction were found to be shikonin, arnebin-1 and *β,β’*-dimethyl acrylalkannin. Among all of these components, arnebin-1 has pro-angiogenic and synergistic effects with vascular endothelial growth factor (VEGF) which further augments the wound healing process (Safavi et al., 2019). Similarly, several other phytoconstituents isolated from *n*-hexane-dichloromethane extract of Onosma argentatum Hub.-Mor. roots i.e. deoxyshikonin, acetylshikonin, 3-hydroxyisovalerylshikonin and 5–8-*O*-dimethylacetylshikonin were found to be effective in treatment of burns wounds. In another study, the efficacy of mixture of olive oil, beeswax and root extract of Alkanna tinctoria Tausch. was examined on burn wounds which showed rapid epithelization and angiogenesis (Gümüş & Özlü, 2017). Moreover, this extract has been established to increase fibroblasts production which amplifies tissue regeneration and provides better perfusion to wound area resulting in granulation tissue formation (Yazdinezhad, Monsef-Esfahani, & Ghahremani, 2013) **(**Fig. 4**).** The healing effects of ointment loaded with *Arnebia euchroma* extract were also compared with standard silver sulfadiazine on second degree burns and the extract demonstrated higher efficacy. Fibroblast proliferation, cell migration and collagen synthesis were observed to be the major mechanisms in its healing process (Nasiri et al., 2016).

Furthermore, the active constituents of *L. erythrorhizon* such as shikonin, isobutyl-shikonin, *β*-hydroxyl-isovaleryl-shikonin and *α*-methyl-*n*-butyl-shikonin were loaded in chitosan/gelatin-based scaffolds and examined for their wound healing potential. The results demonstrated the mechanism of healing via regulation of epithelial-mesenchymal transition (EMT) through TGF-*β* expression **(**Table 1**)** (Hsiao, Tsai, & Chak, 2012, Wang, Kravchuk, & Kimble, 2010, Yao, Chen, Chen, Li, & Huang, 2019).

**Table 1:** Mechanisms involved and pharmacological outcomes from various investigation of alkannin/shikonin containing plant extracts, alkannin/shikonin and its derivatives on different wound models.

Test compounds/extract	Cell cultures/*In vitro*/*In vivo* assays	Mechanism involved/ Pharmacological outcomes	Wound types	References
Shikonin, isobutylshikonin, *β*-hydroxylisovalerylshikonin, *α*-methyl-*n*-butyl-shikonin	Cytotoxicity assay using L929 mouse fibroblasts; *In vivo* wound healing assay	Proliferation of fibroblasts; Synergistic effect of gelatin and chitosan promote granulation tissue formation	Skin wounds	Yao, Chen, Chen, Li, & Huang, 2019
Shikonin	Hypertropic scar derived fibroblasts (HSF) and normal fibroblasts (nHSF) cell lines	Activation of Erk1/2 and p38α/β pathway; Induction of hypertropic scar derived fibroblasts apoptosis	Hpertropic or keloid scars	Xie et al., 2015
Shikonin, *β*,*β*’-dimethylacryl shikonin *β*,*β*’-dimethylacryl alkannin	Murine macrophages (RAW264.7), normal human skin fibroblasts (Hs27), human microvascular endothelial cells (HMEC-1), zebrafish line TG (fli1: EGFP)	Inhibition of LPS-induced NO production thereby promoting tissue regeneration and angiogenesis	Burn wounds	Safavi et al., 2019
Alkannin, shikonin, juglone, *α*-napthoquinone, lapachol, deoxyshikonin, *β*,*β*’-dimethylacrylshikonin, acetylshikonin.	Human dermal scar-derived fibroblasts (HSF) and Human ‘normal’ dermal fibroblasts (nsHSF)	Inhibition of TGF-β1 induced collagen deposition and cell mediated contraction; Phosphorylation of P-Erk and NF-κβ	Dermal scars	Fan et al., 2019
*Alkanna strigose* extract	Excision and incision wound models	Increase in wound contraction rate and promoting granulation tissue formation.	Suppurative wounds	Aburjai, Al-Janabi, Al-Mamoori, & Azzam, 2019
2-methyl-*n*-butrylshikonin, acetylshikonin, isovalerylshikonin, deoxyshikonin	Anti-oxidant activity using DPPH assay and wound healing activity using Linear incision wound model	Accelerative effect on proliferation and migration thereby promoting re-epitheliazation	Incision wounds	Eruygur, Yılmaz, Kutsal, Yücel, & Üstün, 2016
2-bromo-1,4-naphthoquinone, 2-*N*-isonicotinoyl-hydrazide-1,4-naphthoquinone, 1-*N*-Isonicotinoyl-hydrazone-[2hydroxy-3-(3-methyl-2-butenyl)]-1,4-naphthoquinone	Mouse fibroblast cell lines 3T3, MTT assay, Scratch assay, Excision wound model	Inhibition of lysophosphatidic acid signaling pathway and MAPK signaling pathway	Diabetic wounds	Cardoso et al., 2018
Shikonin, acetylshikonin, *β*,*β*’-dimethylacrylshikonin	Excision wound model	Increase in collagen fibre levels in granuloma tissue via expression of TNF-α	Excision wounds	Chen, Yu, Hsu, Tsai, & Tsai, 2018
Deoxyshikonin	Human umbilical vein vascular endothelial cells (HUVECs), immortalized human kerationocytes (HaCaT)	Stimulation of phosphorylation of p38 and extracellular signal regulated kinase.	Full-thickness dermal wounds	Park et al., 2017
*Arnebia euchroma* roots	Randomized, single blind clinical trials	Promote angiogenesis via increased expression of matrix mucopolysaccharide deposition, collagen synthesis and fibroblasts proliferation.	Second degree burns wounds	Nasiri et al., 2016
*Echium arenarium* extract	Murine 218 macrophagic cells (Raw264.7). *Bacillus cereus*, *Listeria monocytogenes, Staphylococcus aureus*, Methicillin-resistant *Staphylococcus aureus* (MRSA), *Enterrococcus faecalis, Escherichia coli, Pseudomonas aeruginosa*, *Klebsiella pneumonia*, *Leishmania major* (GLC94) and *Leishmania infantum* (LV05)	Anti-oxidant, anti-bacterial activity and anti-leishmanial activity	Cutaneous leishamaniatic wounds	Kefi, et al., 2018
*Alkanna tinctoria* extract	Experimental study on patients with second degree burns.	Increased fibroblastic activity and accelerated granulation.	Full-thickness burn wounds	Gümüş & Özlü, 2017

### 2.2 Antimicrobial activity

**Fig. 5:**
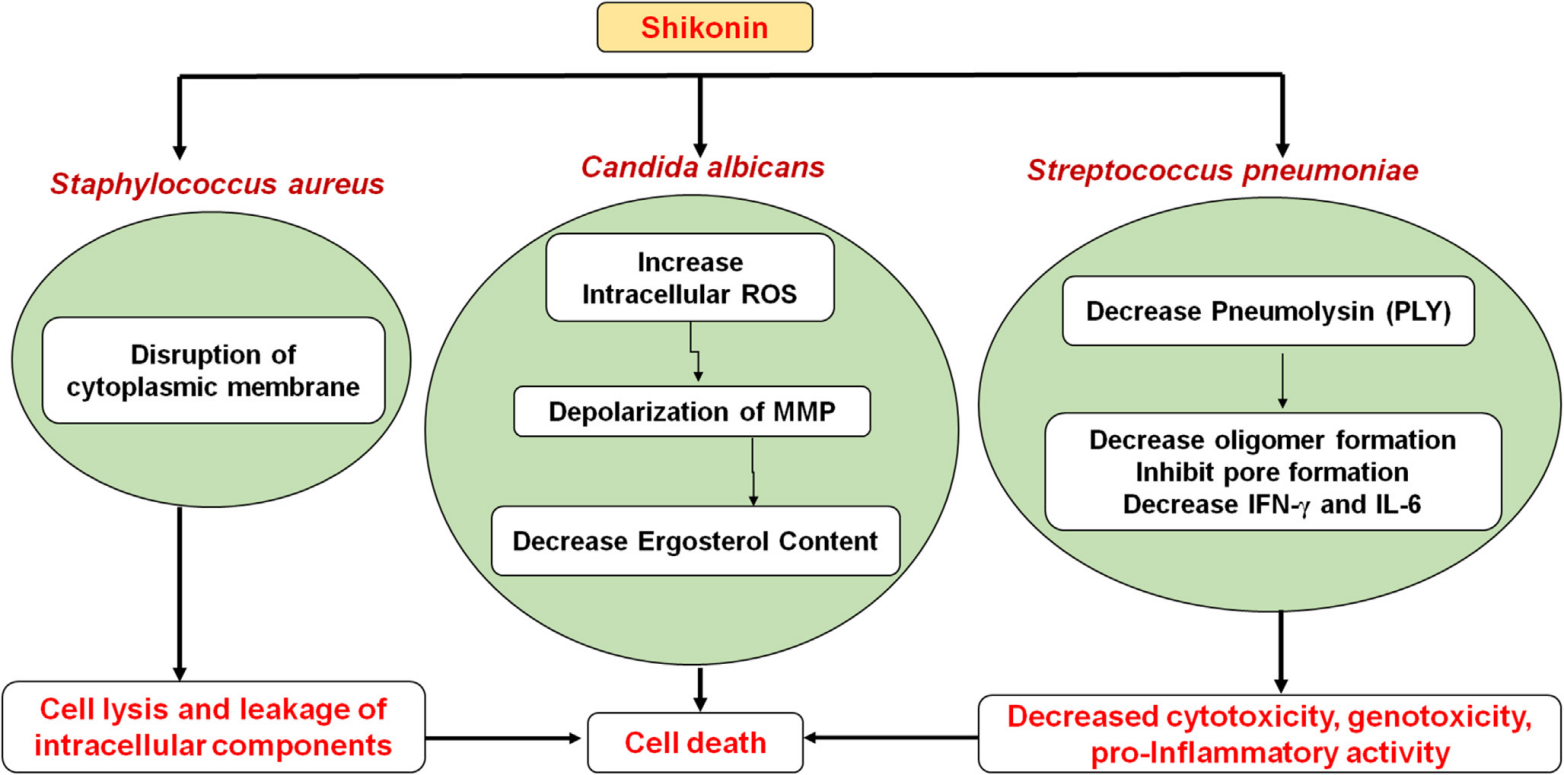
Mechanistic action of shikonin and its derivatives on various microbial strains viz. *Staphylococcus aureus*, *Candida albicans* and *Streptococcus pneumoniae* where ROS is reactive oxygen species and MMP is mitochondrial membrane potential.

Traditional Chinese herb *L. erythrorhizon* has been widely used in treatment of a wide range of infections (Yan, Tan, Miao, Wang, & Cao, 2019). *Candida albicans* is the major opportunistic pathogen and major cause of fungal infections in humans. Shikonin showed significant inhibitory effect on the growth of *C. albicans* through multiple mechanisms. It markedly increases the intracellular ROS (reactive oxygen species) and causes depolarization of mitochondrial membrane potential. It was observed to reduce the ergosterol content also. Further, it could lead to the upregulation of thioredoxin reductase-related gene (TRR1), NADPH oxidoreductase-related gene (EBP1) and mitochondrial respiratory electron transport chain-related gene (MRF1) (Fig. 5**)** (Miao et al., 2012). Moreover, shifting mitochondrial aerobic respiration and promoting endogenous reactive oxygen species augmentation contributes to DNA damage (Liao et al., 2016). On the other hand, *Staphylococcus aureus* is one of the most common and predominant causes of persistent infections in chronic wounds; It contributes the nosocomial infections and hence, proved to be biggest pathogenic burden. Being an adaptable pathogen, it has ability to gain resistance against broad spectrum of antibiotics. Resistance development occurs as a result of horizontal gene transfer (HGT) via transduction, conjugation, or transformation (Craft, Nguyen, Berg, & Townsend, 2019). However, resistance to methicillin and other β-lactam antibiotics is acquired due to *mecA* gene transfer situated on a mobile genomic element, the Staphylococcal chromosome cassette mec (SCCmec) (Deurenberg & Stobberingh, 2009). In order to combat multidrug resistance, shikonin is of utmost importance. The TEM images of shikonin treated Methicillin-resistant Staphylococcus aureus (MRSA) shows disruption of cytoplasmic membrane and cell lysis with subsequent leakage of intracellular components. In addition, shikonin directly binds to peptidoglycan (PGN) which is main component of Gram-positive bacterial cell wall (Lee et al., 2015).

*Streptococcus pneumoniae* is another pathogen causing severe infections in humans. Pneumolysin (PLY) is vital virulence trait of *S. pneumoniae* which possesses cytotoxicity, genotoxicity and pro-inflammatory activity. The treatments for this microbe were limited due to its ubiquitous antibiotic resistance. Shikonin has been found as therapeutically effective for *S. pneumoniae* based pneumonia as it antagonizes the hemolytic activity of PLY thereby reducing the cytotoxicity of PLY. It also inhibits oligomers formation and block pore formation on the cell membrane which leads to decreased production of IFN-γ and IL-6 (Zhao et al., 2017). Moreover, shikonin was also found effective for periodontal diseases as it has ability to inhibit *Porphyromonas gingivalis*, *Fusobacterium nucleatum*, *Streptococcus mutans* and *Lactobacillus acidophilus* which are most susceptible bacterial strains involved in dental caries **(**Table 2**)** (Li, Xu, Zhu, & Wang, 2012). In the latest studies, shikonin and its derivatives including shikonin glucoside, 4-chlorophenylacetyl shikonin, lithospermidin B and Angelyl shikonin were assessed for protein binding with Main protease (Mpro) of SARS CoV-2 revealed shikonin and some derivatives as potential antiviral agent of Covid (Woo & Das, 2022).

**Table 2:** Mechanisms involved and pharmacological outcomes from various antimicrobial investigations on alkannin/shikonin and its derivatives.

Shikonin and its derivatives	Cells/Targeted strains	Mechanism involved	References
Shikonin	Human lung epithelial cells (A549) Murine model of endonasal pulmonary infection; *Streptococcus pneumoniae* strain D39 serotype 2 (NCTC 7466)	Antagonistic effect on haemolytic activity of pneumolysin (PLY); Reduce the cytotoxicity of PLY by inhibiting oligomers formation and blocking pore formation on the cell membrane; Decreased production of IFN-γ and IL-6.	Zhao et al., 2017
	*Aspergillus terreus* (NCCPF860035)	Upregulation of Mpkc, spm1, protein kinase (Pkc-c), protein kinase (dsk1) serine/threonine-protein kinase and small GTPase ras-1 proteins; Moderate increase in cAMP.	Shishodia & Shankar, 2020
	SC5314, SN250 strains	Shifting mitochondrial aerobic respiration and promoting endogenous reactive oxygen species augmentation thereby causing DNA damage.	Liao et al., 2016
	*Agrobacterium rhizogenes* 15834	−	Boehm, Sommer, Li, & Heide, 2000
	*S. aureus* ATCC 33591 (MRSA) and *S. aureus* ATCC 25923 (MSSA)	−	Lee et al., 2015
Iranian *Arnebia euchroma* extract	*Trichophyton mentagrophytes* (PTCC5054), *Microsporum canis* (PTCC5069), *Trichophyton rubrum* (PTCC5143), *Candida albicans* (PTCC5027), *Aspergillus fumigatus* (PTCC5009) and *Penicillium chrysogenum* (PTCC5076)	−	Sabokbar, Tabaraie, Karimi, & Talebi, 2017
*Lithospermum erythrorhizon* seeds	*Bacillus subtilis* 613R, *Clavibacter michigenensis* subsp. nebraskensis CN74-1, *Agrobacterium radiobacter* K84, *Agrobacterium tumefaciens* C58, *Escherichia coli* ESS, *Erwinia carotovora* ATCC 15713, *Pseudomonas aureofaciens*, *Pseudomonas fluorescens*, *Pseudomonas syringae* B, *Ralstonia solanacearum*, and *Serratia marsecens*	−	Brigham, Michaels, & Flores, 1999
Acetylshikoninshikonin, *β*-sitosterol, *β,β*-dimethylacryl shikonin and deoxyshikonin from L. erythrorhizon	*Porphyromonas gingivalis* (ATCC 33277), *Streptococcus mutans* (UA 159), *Fusobacterium nucleatum* (ATCC 25586) and *Lactobacillus acidophilus* (ATCC 4356)	−	Li, Xu, Zhu, & Wang, 2012
*β,β*-dimethyl acrylshikonin, isovaleryl shikonin, *β*-hydroxyiso valerylshikonin and shikonin isovalerate from *Arnebia hispidissima* (Lehm.) DC.	*Escherichia coli* (ATCC-5922), *Klebsiella pneumonie* (ATCC-59008) *Eterobacter cloacae* (ATCC25924), *Bacillus subtilis* (ATCC-10031), *Staphylococcus aureus* (ATCC-25923), *Streptococcus pneumoniae* (ATCC-10032), *Aspergillus niger, Rhizoctonia phaseoli*, *Aspergillus flavus*, *Penicillium chrysogenum* and *Candida albicans*	−	Singh & Sharma, 2012
Shikonin	*Candida albicans* (SC5314)	Increased intracellular ROS and depolarization of mitochondrial membrane potential	Miao et al., 2012
Deoxyalkannin, alkannin, acetylalkannin, Isobutyryl alkannin, *β*-hydroxyiso valerylalkannin, 2′'-(*S*)-*α*-methylbutyryl alkannin, Propionyl alkannin, methyl jasmonate and Teracrylalkannin.	*Staphylococcus aureus* (ATCC 25923), *Escherichia coli* (ATCC 25922), *Staphylococcus epidermidis* (ATCC 12228), *Klebsiella pneumoniae* (ATCC 13883), *Enterobacter cloacae* (ATCC 13047), *Pseudomonas aeruginosa* (ATCC 227853) *Candida albicans* (ATCC 10231), *Candida tropicalis* (ATCC 13801) and *Candida glabrata* (ATCC 28838).	−	Damianakos et al., 2012
Alkannin, shikonin, acetyl alkannin, acetyl shikonin, *β,β*-dimethyl acryloyl alkannin isovaleryl alkannin, and *R*-methylbutyryl alkannin, Cinnamoyl alkannin, 3,4-(methylenedioxy)cinnamoyl alkannin, isobutyryl alkannin from *Arnebia euchroma*	*S. aureus, E. faecalis* and MRSA.	−	Shen et al., 2002

### 2.3 Anticancer activity

Cancer is one of the most fatal diseases and one of the primary causes of deaths globally. The incidence of cancer in India has been expanding in the last two decades as in other developing nations. Not only the incidence but pattern has also changed to a great extent (Ferlay et al., 2010, Jha, 2009, Guddati, 2012, Rocconi et al., 2012). Anticancer drug resistance is another major obstacle in the effective cancer treatment. It is known that conventional anticancer drugs are likely to cause apoptosis. Due to sensitivity to neoplastic cells to apoptosis, they significantly become resistant via antiapoptotic progression and dysregulation of apoptotic machinery (Han et al., 2007). In addition, antiapoptotic progression in neoplastic cells involves overexpression of antiapoptotic proteins (Bcl-2, Bcl-x1, Mcl-1, c-FLIP), proapoptotic proteins mutations (p53, Apaf-1, Bax, FAS) and loss of caspases (Caspase-3 and Caspase-8) which significantly contributes to drug resistance (Bonora et al., 2015). Therefore, defects in the apoptotic signaling and upregulation of apoptotic inducers enormously limit the effectiveness of chemotherapy. Presently, overcoming the drug transporter-mediated resistance is possible as it works on fewer targets whereas apoptosis mediated drug resistance is highly difficult because of multiple potential targets (Han et al., 2007).

**Fig. 6:**
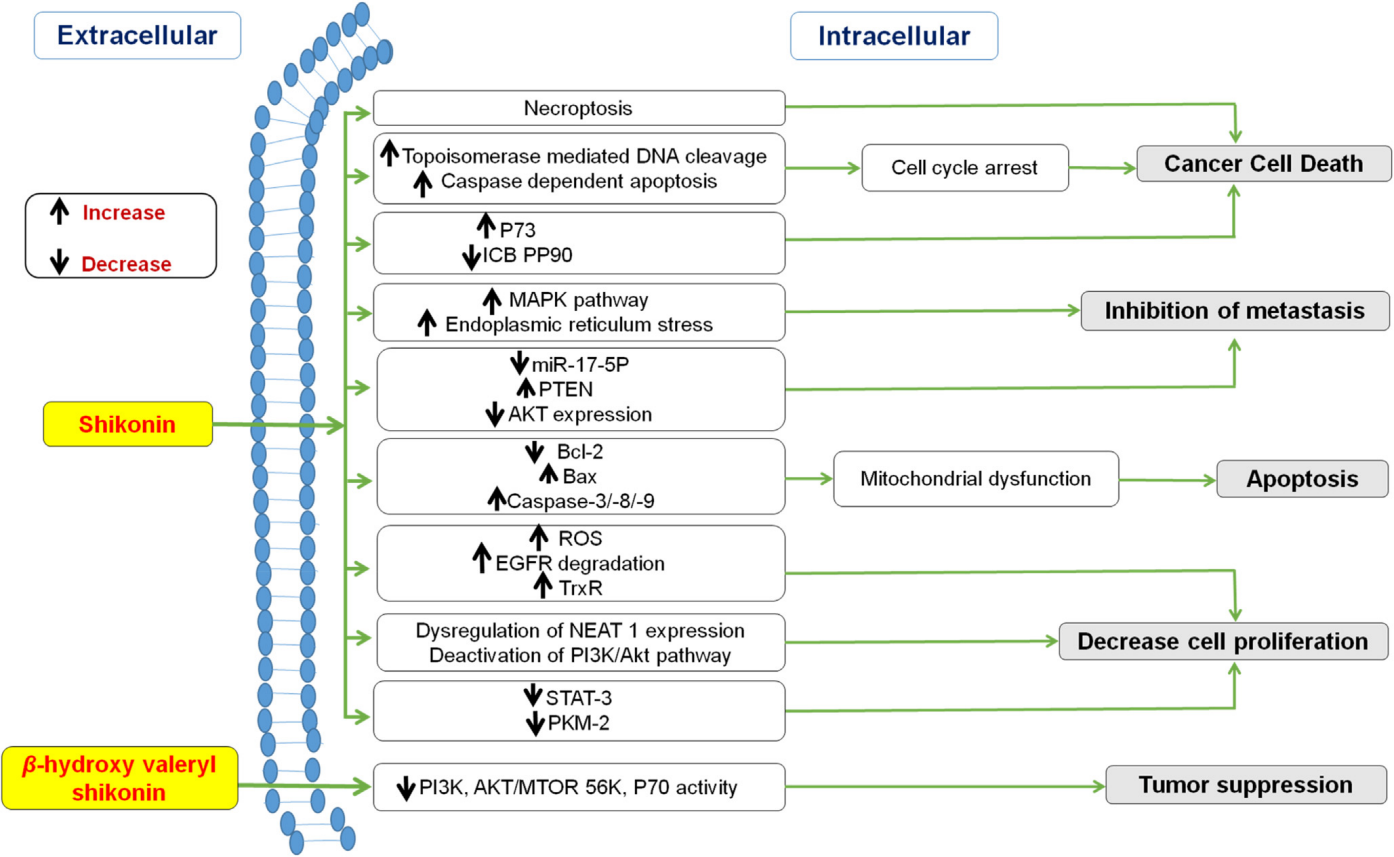
Mechanistic anticancer pathways of shikonin and *β*-hydroxy valeryl shikonin.

Owing to its strong and broad spectrum anti-cancer activity, shikonin and its derivatives are gaining popularity. A study by a research group revealed the necroptotic mechanism of shikonin to promote non-apoptotic cell death (Degterev et al., 2005). Moreover, shikonin could circumvent cancer drug resistance through induction of necroptosis. Necroptosis is a programmed cell death characterized by necrotic cell morphology and activation of autophagy (Han et al., 2007). Also, shikonin promotes topoisomerase mediated DNA cleavage, caspase–dependent apoptosis and cell cycle arrest via activation of tumor suppressor gene p73 and downregulation of ICBP90 **(**Fig. 6**)**. Additionally, p73 is responsible for transcription of various p53 target genes such as p16^INK4A^, PIUMA (p53-upregulated modulator of cell death) and p21 (Jang, Hong, Jeong, & Kim, 2015).

A recent report indicates that ICBP90 is overexpressed in patients with cervical cancer. Cervical cancer is second most malignant tumor in women after breast cancer. Annually, the global rate of cervical cancers is about 60 million cases with 25 million deaths (Kaarthigeyan, 2012). Also, high risk human papillomavirus infection (HPV) long term infection of HPV is the leading cause of cervical intraepithelial neoplasia, precancerous lesions and cervical carcinoma (Cook et al., 2017). Previous studies demonstrated that β-hydroxyisovaleryl shikonin (β-HIVS), a shikonin derivative, possesses inhibitory effect on HeLa cells through apoptosis and prevent tumor cell proliferation. β-HIVS retards PI3K activity and downregulates AKT/mTOR signaling along with reduced P7056K expression levels which ultimately leads to tumor suppression (Lu et al., 2015).

On the other side, breast cancer is most prevalent malignancy in women. Recently, triple-negative breast cancer (TNBC) accounts for about 20% of all new cases of breast cancer accompanied with higher grade and distinct metastatic potential. Therefore, suppression of metastasis might be a promising therapy for TNBC patients (Lambert, Pattabiraman, & Weinberg, 2017, Temian, Pop, Irimie, & Berindan-Neagoe, 2018). Essentially**,** epithelial-to-mesenchymal transition (EMT) plays a pivotal role in regulating metastasis process. EMT involves loss of epithelial phenotypes and the gain of mesenchymal features. It is characterized by downregulation of epithelial cell-surface markers such as occludin, E-cadherin and zonula occludens-1 whereas upregulation of mesenchymal markers such as *N*-cadherin and vimentin. Of particular interest, shikonin has been established as an effective strategy with good therapeutic potential for TNBC patients. It significantly reduces the expression of miR-17-5p which leads to activation of tumor suppressor gene (PTEN). However, overexpression of PTEN downregulates the Akt expression thereby inhibiting metastasis (Bao et al., 2020).

In recent years, the incidence of colon cancer is considerably increasing in western countries attributing to unhealthy lifestyles. The safety and efficacy of shikonin was determined against colon cancer. Studies demonstrated that shikonin promotes cell death via mitochondrial dysfunction which is induced by downregulation of Bcl-2 and upregulation of Bax, Caspase-3 and Caspase-9. In addition, activation of MAPK pathway and increased endoplasmic reticulum stress triggers apoptosis (Han et al., 2019, Liang et al., 2017). Specifically, anti-cancer activity of shikonin against gefitinib–resistant non-small cell lung cancer (NSCLC) was investigated. Shikonin showed strong cytotoxicity against NSCLC cell lines. Also, it effectively generates ROS and stimulates EGFR degradation resulting in inhibition of TrxR thereby inducing apoptosis (Li et al., 2017). Another study on paclitaxel-resistant non-small cell lung cancer, shikonin induces dysregulation of NEAT1 expression which leads to deactivation of PI3K/Akt pathway hence, inhibiting cell proliferation. Simultaneously, shikonin considerably increases expression of PARP and caspase-3 and caspase 9 cleavages (Zang, Rao, Zhu, Wu, & Jiang, 2020). Researchers reported that activation of STAT3 and PKM2 regulates cell proliferation (Cao et al., 2020, Hoshino et al., 2007). Therefore, STAT3 and PKM2 can be considered as key targets for tumor suppression. Recent studies indicated that shikonin markedly reduced the expression of STAT3dimer and PKM2 gene thereby inhibiting inhibits melanoma cell growth (Cao et al., 2020, Liu et al., 2020). Furthermore, the deactivation of NFƙB also contributes in inhibiting cancer-inducing inflammation by decreasing release of inflammatory cytokines such as COX-2, iNOS and IL-6 **(**Table 3**)**. In the recent studies shikonin was tested against Acute Myeloid Leukemia. Shikonin impairs the mitochondrial activity and electron transport chain complex-II to selectively target leukemia cells (Roma et al., 2022). Moreover, inhibitory potential of shikonin was reported on Sunitinib-Resistant renal carcinoma cells. It acts by necrosome complex formation and downregulation of AKT/mTOR signaling pathway (Markowitsch et al., 2022). Lately, shikonin was tested and found effectively active against Mutant-non small lung cancer cells. It induces necrosis and apoptosis of cancer cells via thioredoxin reductase 1 inhibition following SecTRAPs generation and oxygen-coupled redox cycling pathway (Zhang et al., 2022). One of the study demonstrated anticancer effect of shikonin against colon cancer cells. It triggers the apoptosis of cancer cells by checking the cancer cell growth in S phase of cell cycle (Chen et al., 2021). Shikonin is found to be a potential inhibitor in pancreatic cancer as it mediates PD-L1 degradation which in turn suppresses immune evasion in pancreatic cancer cells via NF-κB/STAT3 and NF-κB/CSN5 signaling pathway (Ruan et al., 2021). The anticancer potential of shikonin co delivered with siTGF-β against triple negative breast cancer cells was investigated by Li et al and this co-delivery approach was found to be magnificently efficacious for the same (Li et al., 2022). In a nutshell, shikonin/alkannin and their derivatives are promising candidates for anticancer activity which act by various signaling pathways.

**Table 3:** Mechanism involved and pharmacological outcomes from various anticancer investigations on shikonin and its derivatives.

Compounds	Cell lines/*In vitro*/*In vivo* assay	Mechanism involved	Types of cancer	References
Shikonin	Human normal lung fibroblast cell line CCD19 and human NSCLC cell lines (HCC827, H1650 and H1975)	Induces EGFR degradation causes deactivation of Tyr1173 and Tyr1068 of EGFR; Inhibits TrxR1 to activate ROS-mediated apoptosis	Gefitinib-resistant non-small cell lung cancer	Li et al., 2017
	Human epithelial colorectal adenocarcinoma Caco-2 cells. AOM/DSS model.	Inhibition of COX-2, iNOS and IL-6 via deactivation of NFƙB; Inhibits Bcl-2 and activates Caspase-3	Colon cancer	Andújar, Ríos, Giner, & Recio, 2013
	Normal human colon epithelial cell line (NCM460), well-differentiated colon carcinoma cell lines (HT29 and HCT116), poorly differentiated colon carcinoma cell line (SW480). Nude mouse tumor xenograft model	Overexpression of SIRT2; Inhibits the viability of SW480 cells and arrests the cell cycle at the G2/M stage; Inhibition of ERK1/2 phosphorylation	Colorectal cancer	Zhang et al., 2017
	MCF-7 and SK-BR-3 cells	Downregulation of ERα, GPER, EGFR and p-ERK expressions; Inhibits the proliferation in MCF-7 and SK-BR-3 cells; Arrest cell cycle at G0/G1 phase in MCF-7 and induce apoptosis in SK-BR-3 cells	Breast cancer	Yang et al., 2019
	Human lung cancer cells (A549) Nude mouse tumor xenograft model	Significant increase in RIP1 levels leading to necroptosis	Non-small cell lung cancer	Kim et al., 2017
	Human colon cancer cell lines HCT116, SW480 and human normal colon mucosal epithelial cell line NCM460.	Decreased Bcl-2 and Bcl-xl expression; Increased caspase 3 and 9 activities. Depolarization of mitochrondrial membrane potential	Colon cancer	Liang et al., 2017
	Myelogenous leukaemia cell line (K562cells), breast cancer cell line (MCF-7cells) and cervical cancer cells (HeLa cells) Xenograph tumour model	Irreversible inhibition of human recombinant CDC25 phosphatases; Inhibit dephosphorylation of CDK1 thereby inducing cell cycle arrest at G2/M phase	Cancer	Zhang et al., 2019
	Human lung cancer cells (A549) Nude mouse tumor xenograft model	Suppression of NEAT1 and Akt signaling	Paclitaxel-resistant non-small cell lung cancer	Zang, Rao, Zhu, Wu, & Jiang, 2020
	Breast cancer cell line (MCF-7 cells)	Downregulation of Bax expression and reduced exosomal secretion leading to suppress proliferation	Breast cancer	Wei et al., 2016
	Human colon cancer cell line (SNU-407)Terminal deoxynucleotidyl transferase-mediated digoxigenin dUTP nick-end labeling (TUNEL) assay.	Induces the mitochondrial dysfunction via downregulation of Bcl-2 and upregulation of Bax, Caspase-3 and Caspase-9; Activation of MAPK Pathway. Increased Ca**^2+^** levels leading to ER stress	Colon cancer	Han et al., 2019
	Murine mammary cancer (4 T1) and human breast cancer cells (MDA-MB-231) Orthotopic model of murine mammary cancer cells.	Activation of p38 and JNK signaling pathways; Increase caspase3/7 activity; Inhibits proliferation, migration and invasion ability of cells	Breast cancer	Xu et al., 2019
	Human breast cancer cells (MDA-MB-231)	Reduced expression of miR-17-5p and upregulation of PTEN expression with decreased levels of Akt and p-Akt leads to EMT suppression	Triple negative breast cancer	Bao et al., 2020
	Human ovarian cancer cell (SKOV3)	Downregulation of Bcl-2, AKT and PI3K whereas upregulation of Bax, Caspase-3 and Caspase-9	Ovarian cancer	Zhang et al., 2020
	Human melanoma (A375) and normal human liver-derived cells (MIHA) Zebrafish Tumor Model	Reduced expression of STAT3 dimer. Decreased levels of Bcl-2, Mcl-1, MMP-9 and MMP-2	Malignant melanoma	Cao et al., 2020
	Human colorectal adenocarcinoma (SW620 and HCT116 cell lines) Subcutaneous tumor mouse model	Induce autophagosome formation via LC3 cleavage; Upregulate expression and promote galectin-1 dimerization	Colorectal carcinoma	Zhang et al., 2020
	Hepatocellular carcinoma cells lines (LM3, SMMC-7721, Huh-7, and HepG2) and a normal liver cell line (LO2)	Downregulates the expression of PKM2; Increased expression of Bax, cyto C, cleaved Caspase-9, and cleaved Caspase-3, and decreased expression of Bcl-2	Hepatocellular Carcinoma	Liu et al., 2020
	Hepatocellular carcinoma cell lines (Huh-7 and HepG2)	Modulation of the SMAD7/TGF‐β signaling pathway through regulation of miR‐106b	Hepatocellular carcinoma	Li & Zeng, 2020
Shikonin, acetyl shikonin, and *β*,*β*-dimethyl acryl shikonin	Human breast cancer cells (MDA-MB-231) and Murine mammary cancer (4T1).	Shikonin was found most potent against TNBC cell lines; Upregulates E-catherin levels whereas downregulates *N*-cadherin and vimentin levels; Stimulate *β*-catenin degradation through enhanced GSK-3β levels	Triple-negative breast cancer	Chen et al., 2019
Shikonin, Deoxyshikoninand *β*,*β*-Dimethyl acrylshikonin	Human amelanotic malignant melanoma cell line A375 (CRL1619), mouse metastatic melanoma cell line B16-F10 (CRL-6475) and mouse nonmetastatic melanoma cell line B16-F0 (CRL6322),	Induces p53-mediated cell cycle arrest;Stimulates ROS -mediated Endoplasmic Reticulum (ER) stress. Activation of p-ERK, p-p38, Caspase-3 and Caspase-9	Skin cancer	Ng, Upton, Leavesley, & Fan, 2020

### 2.4 Miscellaneous activities

**Fig. 7:**
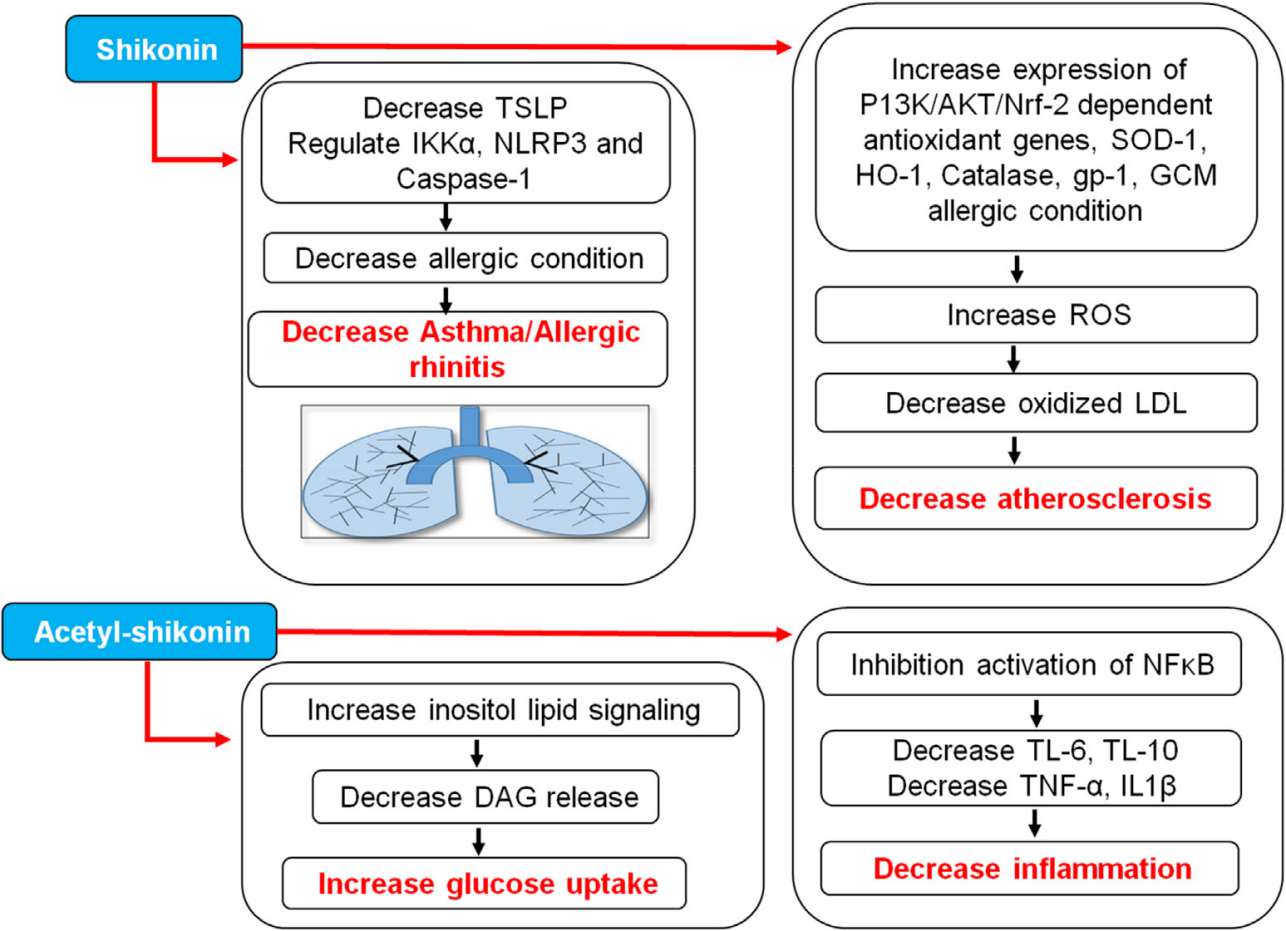
Mechanistic activity of shikonin and acetyl shikonin for treatment of asthma, arthrosclerosis, diabetes and inflammation. LDL, low density lipoprotein; DAG, diacylglycerol; ROS, reactive oxygen species; TSLP, thymic stromal lymphopoietin; IL, interleukins; GCM, global compact on migration.

Apart from pharmacological activities discussed above alkannin/shikonin and their derivatives also possess therapeutic potential against phytogenotoxicity, bronchial asthma, peptic ulcer, spasmogenicity, atherosclerosis, inflammatory diseases, ischemic heart diseases, cataract, hepatotoxicity and impotency (Fig. 7**)** (Yildirim, 2020). Onosma, the biggest genus of Boraginaceae family, is being used as traditional medicine since centuries (Davis, 1970). Shikonin and its derivatives have also been reported to inhibit oxidized low-density lipoprotein (LDL) induced monocyte adhesion by deactivation of NFƙβ and hence used in treatment of atherosclerosis. It is well known that oxidized LDL plays a key role in thrombosis, endothelium apoptosis and vascular smooth muscle proliferation. In addition, it also stimulates release of inflammatory mediators such as cytokines and reactive oxygen species. Moreover, activation of NFƙβ further upregulates the expression of intracellular adhesion molecule (ICAM-1), E-selectin, vascular cell adhesion molecule and monocyte chemotactic protein-1. Hence, the accumulation of oxidized low-density lipoprotein (oxLDL) and inflammatory cells lead to atherosclerosis. Shikonin has also been found effective in retarding oxLDL mediated ROS production through induction of expression of PI3K/Akt/Nrf 2- dependent antioxidant genes such as SOD-1, HO-1, Catalase, GPx-1, GCLM, and GSR (Huang et al., 2015).

Furthermore, the oxidative stress is the major cause of various other medical conditions such as ageing, diabetes, stroke, neurodegenerative disorders, cancer etc. Oxidative stress is often accompanied with higher blood sugar levels. The skeletal muscle cells are rich in insulin-sensitive glucose transporters named as glucose transporter 4 (GLUT4). Their main function is translocation of glucose from cytoplasm to cell membrane aiding in glucose uptake. Therefore, it plays imperative role in regulation of homeostasis of glucose**.** However, the contraction-induced release of reactive oxygen species (ROS) and activation of AMP activated protein kinase (AMPK) may also lead to increased glucose uptake in skeletal muscle cells (Mao, Yu, Li, & Li, 2008, Su et al., 2016). Subsequently, acetylshikonin-induced glucose uptake was significantly inhibited by reduction of PLC-β3 in L6 myotubes, which makes it evident that acetylshikonin-induced glucose uptake may be triggered by activation of inositol lipid signaling and increased DAG release (Huang et al., 2019). On the other hand, ageing is also considered as biggest cause of Alzheimer’s disease. Various studies have shown that oxidative stress, neuronal apoptosis and neuroinflammation plays critical role in pathogenesis of Alzheimer’s disease (Heneka, 2015). SIRT1 is essentially involved in cognitive functions and shows protective effect against aging-related neuronal degeneration. Thus, SIRT1 can be the most promising therapeutic target for Alzheimer’s disease. Multiple studies reported that chronic inflammation associated with raised levels of pro-inflammatory mediators such as IL-6, IL-10, TNF-α and IL-1β. Notably, acetylshikonin reduced the levels of these mediators via inhibiting the activation of NFƙβ and thereby reducing inflammation. Simultaneously, it also inhibits the activation of p21/p53 signaling pathway (Chang et al., 2015). Furthermore, overexpression of thymic stromal lymphopoietin (TSLP) is a major factor contributing to allergic diseases such as asthma, allergic rhinitis etc. Epithelial cell-derived TSLPs control the allergic condition via regulating the activation of *T*-cells, mast cells, and dendritic cells. The findings of the study elucidated that shikonin as well as L. erythrorhizon aqueous extract was able to downregulate TSLP production as well as markedly attenuated the levels of IKKα, NLRP3 and Caspase-1 (Yen et al., 2017). Besides having multiple pharmacological effects, naphthoquinones are also considered as potent allelochemicals as they hold good potential to defend against predators. Previous studies demonstrated that juglone, 1,4-naphthoquinone, plumbagin and 2-methoxy-1,4-naphthoquinone showed anti-feedant activity against the cabbage looper Trichoplusia ni (Akhtar, Isman, Niehaus, Lee, & Lee, 2012). Napthoquinones were also found effective against the dry bean pests Epilachna varivestis and Acanthoscelides obtectus (Cespedes et al., 2016). Moreover, the extreme toxicity of juglone against *Myzocallis walshii* and plumbagin against *Tetrany chusurticae*, *Myzus persicae* and *Illinoia liriodendri* were also investigated. These studies substantiate that juglone and plumbagin are effective insecticidal and acaricidal agents. The inhibitory and toxicity potential of *Onosma visianii* roots against *Spodoptera littoralis* was also investigated. The main active constituents of *O. visianii* roots include isovalerylshikonin and isobutyrylshikonin. Being highly lipophilic in nature, these active moieties easily enter the insect exoskeleton and hinder the physiological processes. Moreover, the ester groups of these moieties increases cuticle penetration via linkage with hydroxyl groups and significantly inhibits acetylcholinesterase (AChE) enzymes. Additionally, it leads to inhibition of mitochondrial respiration thereby controlling larval growth **(**Table 4**)** (Akhtar, Isman, Lee, Lee, & Lee, 2012, Pavela, 2013).

**Table 4:** Mechanism involved and pharmacological outcomes from miscellaneous investigations on alkannin/shikonin containing plant extracts, alkannin/shikonin and its derivatives.

Test compounds	Cell lines/*In vitro*/*In vivo* assay	Mechanism involved	Disease targeted	References
Lithospermum erythrorhizon and Angelica sinensis extract	Human Bronchial Epithelial cell line (BEAS-2B)	Anti-inflammatory effect in Der-p2-stimulated BEAS-2 β cells; Inhibition of TSLP production and suppression of IKKα, caspase-1 and NLRP3	Allergic diseases such as asthma, atopic dermatitis and allergic rhinitis	Yen et al., 2017
*Onosma tauricum* extract	Anti-oxidant assays (DPPH, CUPRAC, ferrous ion chelating, FRAP, polumolybednum, ABTS) and Enzyme inhibitory assays (AChE, *α*-amylase, BChE, tyrosinase, *α*-glucosidase)	Anti-oxidant and enzyme inhibitory activity	Evaluation of antioxidant and enzyme inhibitory potential	Kirkan et al., 2018
*Onosma sieheana* and Onosma stenoloba extracts	Total phenolic assay, total flavonoid assay, anti-oxidant assay, tyrosinase assay, *α*-amaylase assay.	Anti-tyrosinase activity. Increased expression of p-Erk1/2 and reduced extression of tyrosinase related protein 1 and 2	Diabetes	Sarikurkcu, Sahinler, Ceylan, & Tepe, 2020
*Lithospermum radix* aqueous extract	Sub-acute oral toxicity	Suppression of spinal inflammation	Chemotherapy induced neuropathy.	Kim et al., 2019
Shikonin	PAI-1 activity assay, Clot lysis assay, mouse arterial thrombosis model, Mouse liver fibrosis model.	Inhibition of plasminogen activator inhibitor-1 activity; Anti-thrombotic and anti-fibrotic effect	Fibrinolysis	Han et al., 2016
	Human endothelial cell line derived from human lung carcinoma cells and human umbilical vein endothelial cells	Induction of expression of PI3K/ Akt/Nrf 2- dependent antioxidant genes such as SOD-1, HO-1, Catalase, GPx-1, GCLM and GSR; Inhibition of oxLDL-induced intracellular ROS accumulation via NF-ƙβ adhesion	Atherosclerosis	Huang et al., 2015
	Human umbilical vein endothelial cells (HUVEC), human fibroblast-like synoviocyte (HFLS).Collagen-induced arthritis model. *In vivo* chick chorioallantoic membrane (CAM) assay. *Ex vivo* rat aortic ring assay	Inhibition of pro-angiogenic mediators such as TNF-α, IL-12, TGF-β, IL-6, IL-8, VEGF, PDGF,IL-17A and MMPs; Increase expression of Treg/Th17 by deactivation of TLR4/MyD88 pathway	Rheumatoid arthritis	Liu et al., 2020
Isovaleryl shikonin and isobutyryl shikonin	Acute toxicity, chronic toxicity, growth inhibition, antifeedant activity, AChE inhibitory activity and antioxidant assay.	Inhibition of AChE enzymes; Inhibition of mitochondrial respiration thereby inhibiting larval growth	Inhibition of larval growth of Tobacco cutworm *Spodoptera littoralis*.	Sut et al., 2017
Acetylshikonin	Behavioral testing (Morris Water Maze test)	Inhibition of activation of p53/p21 signaling pathway; Upregulation of SIRTI in hippocampus; Anti-apoptotic activity in neuronal cells and attenuated H_2_O_2_ induced oxidative stress	Alzheimer’s disease	Li, Zeng, Su, He, & Zhu, 2018
	L6 rat skeletal muscle cells. Alloxan-induced type I diabetic models.	Activation of p2C-β3/PKCδ cascades via activation of inositol lipid signaling and increase in DAG release	Diabetes.	Huang et al., 2019
Acetylshikonin and isobutyryl shikonin	Anti-genotoxic properties (Umu-test) and cytotoxicity assay (Lung fibroblast cell line (V79)	Inhibition of p450 enzymes, Free radical scavenging activity and anti-genotoxic activity	Carcinogenesis	Skrzypczak et al., 2015

## 3 Isolation and analytical aspects of A/S and their derivatives

A/S and their derivatives have been reported to be isolated from various Boraginaceae family plants **(**Table 5**)** amongst which *L.* erythrorhizon (Lee et al., 2016, Rajasekar et al., 2012, Han et al., 2007, Azuma et al., 2016) and Alkanna tinctoria Tausch. (Mohammed, 2016, Rashan et al., 2018, Jaradat et al., 2018) yield high content of shikonin and alkannin derivatives, respectively. Adding on, the petroleum ether and chloroform fraction of dried roots of L. erythorrhizon elute β,β-dimethylacrylshikonin, isobutyl shikonin, shikonin, 5,8-dihydroxy-2- (-1-methoxy-4-methyl-3-pentenyl) – I, 4 naphthalene dione and – sitosterol, mixture of caffeic acid esters when subjected to column chromatography (Han et al., 2007). On the other hand, column chromatography of powdered roots of A. tinctoria led to elute alkannin, angenylalkannin, 5-methoxy angenylalkannin, alkanfuranol, alkandiol, acetylalkannin whereas dimethylacrylshikonin was obtained using reverse phase column chromatography (Tung, Du, Yuan, Shoyama, & Wang, 2013). On similar lines, Lithospermum euchroma when subjected to column chromatography resulted in the production of acetyl shikonin and β,β’-dimethylacylshikonin (Cheng et al., 2008). Moreover, chiral column in HPLC was used to separate out the enantiomeric excess from the mixture of A/S obtained from dried (under reduce pressure with aid of P2O5) chloroform residue of L. erythrorhizon (Azuma et al., 2016). Furthermore, percolation and soxhlet extraction was used sequentially followed by thin layer chromatography to obtain arnebin-1, arnebin-2, arnebin-3, arnebin-4, arnebin-5, arnebin-6 arnebin-7, tiglic acid, arnebinone, alkannin, arnebinol, cycloarnebin-7, *β*,*β*-dimethylacryl shikonin, isovaleryl shikonin, *β*-hydroxyisovaleryl shikonin and shikonin isovalerate from the powdered roots of *Alkanna hispidissima* (Singh & Sharma, 2012, Yusufoglu et al., 2018). Percolation technique was also used to obtain acetyl alkannin and *β,β*-dimethylacryl alkannin from the viscous red residue of Arnebia nobilis Reichb. f. (Mohapatra et al., 2016). In another report, solid liquid extraction and HPLC-VIS technique was used to elute angelylshikonin, 2-methyl-*n*-butyrylshikonin, and isovaleryl shikonin from the dried roots of Echium etalicum (Albreht, Vovk, Simonovska, & Srbinoska, 2009). In addition to the conventional methods of extraction and isolation, a novel method called supercritical CO_2_ method was used to isolate A/S from the powdered roots of *A. tinctoria*. Supercritical CO_2_ functions as non-polar, lipophilic solvent with alkannin/ shikonin. It was reported that highest yield was obtained at higher temperature and lower flow rates (Akgun, Erkucuk, Pilavtepe, & Yesil-Celiktas, 2011). Subsequently, the ultrasonication technique was also exploited to primarily obtain deoxyshikonin and other naphthoqimone derivatives from the residual extract of Lomandra hastilis (Park et al., 2017, Utkina & Pokhilo, 2017). On the similar lines, L. erythrorhizon extract was subjected to sonication to obtain acetylshikonin, shikonin, deoxyshikonin, β-sitosterol and β,β-dimethylacrylshikonin (Li, Xu, Zhu, & Wang, 2012). Furthermore, Microwave assisted extraction/isolation followed by reverse phase chromatography was used to obtain shikonin and its derivatives from the dried roots of *L. erythorrhizon*. Moreover, the Sephadex column has also been used in one of the reports where *L. erythrorhizon* was taken to elute isobutyrylshikonin (Park, Woo, Kim, Choi, & Park, 2020, Yen et al., 2017). Lately, the higher yield of acetylshikonin in Echium plantagineum has been reported by overexpression of cloned EpGHQH1 (geranylhydroquinone 3″-hydroxylase candidate gene) (Fu et al., 2021). Also, the production of alkannin and shikonin was found to be increased in hairy roots of A. tinctoria when introduced with bacteria belonging to Chitinophaga sp., Allorhizobium sp., Duganella sp., and Micromonospora sp. (Rat et al., 2021).

**Table 5:** Various investigations carried out for analysis of alkannin/shikonin and its derivatives.

Plant	Extraction process	Solvent systems	Methods	Constituents	References
*Lithospermum erythrorhizon*	Sonication	Gradient elution: Petroleum ether-ethyl acetate, petroleum ether - dichloromethane, petroleum ether - acetone, and petroleum ether - ethyl acetate and acetone	Silica gel column chromatography	Acetylshikonin, shikonin, deoxyshikonin, *β*-sitosterol and *β,β*-dimethylacrylshikonin	Li, Xu, Zhu, & Wang, 2012
	Solid liquid extraction	–	Open column of silica gel chromatography	Shikonin, acetylshikonin,5,8-dihydroxy-1.4-naphthoquinone (DH), 1,4naphthoquinone (NAP) and *β,β’*-dimethylacylshikonin.	Cheng et al., 2008
	Solid-liquid extraction	*n*-hexane/2- propanol (90:10, volume percentage)	Chiral HPLC	Shikonin (an improved method)	Azuma et al., 2016
	Maceration	50% hexane in CH_2_Cl_2_, CH_2_Cl_2_, 5% and 33% acetone in CH_2_Cl_2_, and 5% and 33% methanol in CH_2_Cl_2_	Silica gel column chromatography and Sephadex column with methanol.	Isobutyrylshikonin	Park, Woo, Kim, Choi, & Park, 2020
	Maceration	0.085% H_3_PO_4_ buffer and acetonitrile: 10%–25% for 20 min; 25%–70% for 30 min; 70%–90% for 40 min; 80%–90% for 60 min; and 100% for 65 min.	Reverse phase column chromatography	Shikonin, bhydroxyisovalerylshikonin, acetylshikonin, *β*-acetoxyisovalerylshikonin, deoxyshikonin, isobutyrylshikonin, *β,β*-dimethylacrylshikonin, and methyl-*n*-butyrylshikonin	Yen et al., 2017
	Ultrasonic extraction.	Methanol and water with 0.5% acetic acid.	Reverse phase column chromatography	Deoxyshikonin	Park et al. (2017)
*Onosma visianii*	Soxhlet extraction	methanol and water (0.1% formic acid) (90:10)	Semipreparative HPLC	Isovalerylshikonin, isobutyrylshikonin,acetylshikonin, hydroxyisovalerylshikonin, shikonin-*β,β*-dimethylacrylate, propionylshikonin, 5,8 dimethoxy acetylshikonin, 1-(5,8-dimethoxy-1,4-dioxo-1,4-dihydronaphthalen-2-yl)- 4-methylpent-3-en-1-yl 2-methylbutanoate, 5,8 -dimethoxy isobutyrylshikonin, 5,8-*O*-dimethyldeoxyshikonin, 2-(4-hydroxy-4-methylpent-2-en-1-yl)-5,8-dimethoxynaphthalene-1,4-dione.	Sut et al., 2017
*Alkanna strigosa*	Soxhlet extraction	(CHCl_3_: MeOH: H_2_O) (5:4:1)	Preparative tlc	Alkannin and shikonin	Aburjai, Al-Janabi, Al-Mamoori, & Azzam, 2019
*Echium italicum*	Maceration	Hexane-Etilacetae	silica gel column chromatography	2-Methyl-*n*-butyrylshikonin, isovalerylshikonin, acetylshikonin and deoxyshikonin	Eruygur, Yılmaz, Kutsal, Yücel, & Üstün, 2016
*Lomandra hastilis*	Sonication	*n*-hexane–acetone (3:1)	Preparative TLC	5,8-Dihydroxy-2-ethyl-3,6,7-trimethoxy-1,4- naphthoquinone, lomazarin, 2-(1′- acetoxyethyl)-5,8-dihydroxy-3,6,7-trimethoxy-1,4-naphthoquinone, 5,8-dihydroxy-3,6,7-trimethoxy-2-(1′-methoxyethyl)-1,4- naphthoquinone, isonorlomazarin, 5,8- dihydroxy-2-(1′-hydroxyethyl)-1,4-naphthoquinone, 2-(1′- acetoxyethyl)-5,8-dihydroxy-1,4-naphthoquinone, 5,6,8- trihydroxy-2-ethyl-3,7-dimethoxy-1,4-naphthoquinone and ethylmompain dimethyl ether.	Utkina & Pokhilo, 2017
*Alkanna tinctoria*	Extraction with 95% EtOH	• Hexane–EtOAc (20:1–0:1, volume percentage) • Methanol • Hexane–EtOAc (5:1– 4:1, volume percentage)	• Silica gel column chromatography Sephadex column • Reverse phase column chromatography	Angelylalkannin, 5-methoxy angenyalkannin, alkanfuranol, alkandiol, acetylalkannin and dimethylacrylshikonin.	Tung, Du, Yuan, Shoyama, & Wang, 2013
	supercritical CO_2_ extraction	0.025% aqueous TFA and acetonitrile	HPLC-PDA analysis	Alkannin/shikonin	Akgun, Erkucuk, Pilavtepe, & Yesil-Celiktas, 2011
*Echium italicum*	Solid–liquid extraction	MeOH:HCOOH (20:1, volume percentage) and THF:MeCN:H_2_O:HCOOH (30:20:50:0.5, volume percentage)	Chiral thin-layer chromatography and semi-preparative HPLC	Angelylshikonin, 2-methyl-*n*-butyrylshikonin, and isovaleryl shikonin	Albreht, Vovk, Simonovska, & Srbinoska, 2009
*Arnebia nobilis* Reichb.f.	Percolation	–	Silica gel chromatography	Acetyl alkannin, acetoxyisovaleryl alkannin and *β,β* dimethylacryl alkannin	Mohapatra et al., 2016
*Alkanna hispidissima*	Percolation	Hexane - acetone - acetic acid	Thin layer chromatography	Arnebin-1, arnebin-2, arnebin-3, arnebin-4, arnebin-5, arnebin-6 arnebin-7, tiglicacid, arnebinone, alkannin, arnebinol, and cycloarnebin-7.	Yusufoglu et al., 2018

## 4 Patent applications

Forecasting the market potential, numerous patent applications on inventions containing alkannin/shikonin and its derivatives have been filed by various research groups across the world. Brief details of these applications are divided into two categories viz. therapeutic and analytical and are summarized in Table 6, Table 7**.**

**Table 6:** Therapeutic patents of alkannin/shikonin and their derivatives.

Titles	Targeted diseases	Mechanism of action	References
Acylatedalkannin or shikonninderivs.- useful as dermatological, bactericidal and fungicidal medicaments	Treatment of skin lesions: ulcers, burns, wounds, scruf, skin cancers	Antibacterial and anti-inflammatory effect	Papageorgiou, 1980
Process for preparing arnebia root medicine with broad-spectrum medical functions	Measles, rashes, ulcer sores, eczema, burns	Proliferation of fibroblasts	Song, 2004
*Alkannin* derivatives as immune inhibitors and metal complexes thereof	Arthritis, scleredema, lupus erythematosus, HIV infection and malignant tumor	Immunological suppression of chemokines amd HIV-type 1	Li & Hu, 2004
Use of alkannin in preparing medicine for treating tumor disease	Treatment of tumor, effective on eh tumour, effective on medicine resisting tumor cells	Killing tumor cells with *p*-glycoprotein	Hu & Fang, 2005
Application of shikonnin in preparing medicine for inducing apoptosis	Treatment of tumor	Shikonin induces ROS production and cytochrome *c* release in cancer cells.	Hu & Han, 2007
Application of Xinjiang radix macrotomiae for treating flatwart, common wart and fig wart	Treatment of verrucous disease, Flat wart, common wart, fig wart	Diminishing the inflammation of hurt on an afflicted part, healing of hurt without leaving scar	Li & Chen, 2009
Method of treatment of virus infections using shikonnin compounds	Virus infections, mycoplasma infections, malignant tumor	Promoting idiosyncratic cell mediated immunity and improves immune response of *T*-lymphocytes	Wang, 2008
Antineoplastic sulphur-comtaining alkannin and naphthoquinone derivatives	Antineoplastic	Inhibition of tumor cell growth	Li, Zhao, Xei, He, & Guo, 2008
Antineoplastic alkannatinctoria ketoximes derivatives	Antineoplastic	Retard tumor cell growth	Li & Zhao, 2010
Application of alkannin in preparation of pyuruvate kinase inhibitor	Psoriasis, herpes simplex keratitis	Inhibition of PMK2 activity	Hu, 2011
Medical application of radix arnebiaeseulithospermi naphthoquinone compounds	Crohn’s disease	Inhibition of NF-kβ and STAT-3	Liu & Fan, 2014a
Medical application of gromwell naphthoquinone compounds	Ulcerative colitis	Inhibition of COX-2 and cytokines (INF-γ and IL-6)	Liu & Fan, 2014b
Medical application of lithospermumnahthoquinone compounds	Chronic obstructive pulmonary disease (COPD)	Inhibition of PDE-4	Liu & Fan, 2014c
Pharmaceutical composition for treating flatwart and verruca vulgaris and prepation method for pharmaceutical composition	Flat wart, verruca vulgaris	Resisting inflammation, killing viruses and realizing quick apoptosis of skin vegetation cells.	Yuan & Wang, 2016
Compound traditional Chinese medicine for preventing and treating stigmatosis of freashwater fish	Stigmatosis	Inhibition of influenza virus, gram positive and gram negative bacteria	Liu, Zhao & Wang, 2017
Compositions for metabolic disorders comprising alkannin as an active ingredient	Obesity, hyperlipidemia, fatty liver	Activation of AMPK (AMP activated protein kinase)	Yoon, Lee, Jang, & Jeong, 2017
Application of alkannin in preparation of medicine for treating upper and lower respiratory tract allergic disease	Allergic rhinitis and allergic asthma	Activation and differentiation of TH cells and cytokine secretion	Liu & Yu, 2017
Hydroxynaphthoquinone compounds for treatment of non-small cell lung cancer	Non– small cell lung cancer	Inhibition of EGFR kinase activity and induction of apoptosis in cancer cells	Liu, Leung, Li, & Fan, 2018
Herqueiazole-containing medicine for controlling inflammation	Inflammation	Synergistic effect of shikonin and herqueiazole	Zhuang & Zhang, 2017
External biological preparation for feminine vagina prophylaxis and health-care as well as treatment of gynaecological genital tract inflammation, and preparation method	Cervical erosion, vaginitis, pelvic inflammation	Eliminating vaginal bacteria and maintaining vaginal flora and acid base balance	Wang & Chen, 2017
Omeprazole enteric-coated capsules capable of inhibiting gastric acid secretion	Gastric and duodenal ulcer	Inhibition of H^+^, K^+^ ATPase enzyme activity	Li, 2018
Composition for treating burns and scalds	Treating burns and scalds.	Antibacterial and antiinflammatorey effect	Liu, Wei, Zhong, Zuo, Yi, & Yang, 2017
Shikonin and derivant thereof are as the application of gene therapy sensitizer	Cancer	Inhibition of TNF-ᾳ	Ling, Wang, Wang, & Zhang, 2017
Externally-applied anti-inflammatory agent containing radix lithospermii extract	Inflammation	Inhibition of STAT 3 (Signal transducers and activators of transcription) pathway	Chu, 2018a, Chu, 2018b

**Table 7:** Analytical/Biosynthetic patents of alkannin/shikonin and their derivatives.

Patent No./ Filling date	Plants	Titles	Conditions	Methods of extraction	Compounds	References
CN1079239C (12-04-1995)	Comfrey roots	Gromwell prepn. With wide medical effect and its prepn. Process	Refined oil	Decoction	Shikonin	Song, 2002
CN1117525A (13-06-1995)	*Alkanna tinctoria* shoots	*Arnebia euchroma* (Royle) Johnst. Cell cultivation and prodn. Process by solid two step method	AG-7 growth culture medium and AP-5 germ culture medium	Cell suspension culture	Deoxyshikonin, acetylshikonin, *β*,*β*- dimethyl acrylamide shikonin, dimethylpenteneshikonin	Ye, Li, Song, & Chen, 1996
CN1253972A (29-11-1999)	*Lithospermum officinale* roots	Alkannia and its extraction method	Liquid CO₂	Super critical CO₂ extraction	Shikonin, acetylshikonin, dimethyl acrylamide shikonin, *β* hydroxyl isovalerylshikonin, 2,3 dimethyl pentene shikonin.	Xu, Wang, & Huang, 2000
CN1384149A (17-05-2002)	Comfrey roots	Gromwell haematochrome extracting process	Liquid CO₂	Super critical CO₂ extraction	Alkannin	Wang, 2002
CN1633841A (26-12-2003)	*Arnebia euchroma*	Method for promoting Xinjiang alkannatinctoria callus growth using rare earth element	N₆ solid medium	Callus growth culture	Shikonin	Wang, Fang, & Wang, 2005
CN1546450A (08-01-2004)	Dried *Arnebia* roots	Preparation method of high purity alkannaphthaquinone	Supercritical CO₂	Super critical CO₂ extraction	Alkannin	Li & Hu, 2004
CN101434530A (12-12-2008)	Comfrey dried purple roots	Method for extracting alkannin from alkanet	Ethanol	Solid liquid extraction	Shikonin	Zu et al., 2009
CN101942212A (15-07-2010)	Comfrey powder	Method of extracting alkannin naphthoquinone pigment	1,1,1,2- Tetrafluoro ethane	Molecular distillation	Dimethyl acrylamide shikonin, isovalerylshikonin	Liu & Liu, 2011
CN101906028B (26-08-2010)	Comfrey roots powder	Method for extracting benzoquinone compound in lithospermum	*n*-Hexane or petroleum ether	Multiple reflux extraction	Alkannin	Yan, Xu, Yu, & Lei, 2013
CN102228499A (20-06-2011)	*Arnebia* roots	Method for separating naphthoquinone active ingredients from sinkiangarnebia root	Petroleum ether, ethylacetate	Ultrasonic extraction	Deoxyshikonin, acetyl shikonin, shikonin, *β*,*β*’-metho acryloyloxyshikonin, isobutyrylshikonin, *β* hydroxyl isovalerylshikonin	Yuan & Yuan, 2011
CN103373913A (15-04-2012)	Comfrey purple grass powder	Extraction method of alkannin	Cyclohexane	Maceration	Shikonin	Pan, Wang, Tang, Li, Wang, & Zhou, 2013
CN103664566A (02-12-2013)	Comfrey purple grass	Alkannin extraction device	Petroleum ether	Ultrasonic crusher extraction	Shikonin	Tang, Wang, & Zhou, 2014
CN105949045A (28-07-2014)	*Arnebia roots*	Method for extracting alkannin from arnebia roots	Supercritical CO₂	Super critical fluid extraction	Shikonin	Guo, Zhang, & Xu, 2016
CN105348065A (04-12-2015)	*Lithospermum erythrorhizon*	Preparation method for high-purity alkannin from lithospermumerythrorhizon	Petroleum ether	Percolation	Shikonin	Yang & Yang, 2016
CN104774151A (30-01-2015)	*Lithospermum mongolia*	Preparation technology of mount taishan *Radix Lithospermi* naphthoquinone active monomers	Petroleum ether, hexanoicacid Capro lactone Hexylalcohol-water.	High performance counter current chromatography	Isopentyl shikonin, hexylshikonnin, isobutyl shikoin	Lei, Haiwei, Jiang, Zhai, Yi, & Jiang, 2015
CN107151203A (03-03-2016)	*Arnebia euchroma*	Method for separating and preparing natural naphthoquinone compounds	*n*-Hexane, ethylacetate, acetonitrile, water	High speed counter current chromato Graphy	Deoxyshikonin, propionyl shikonin, β,β dimethylacryl shikonnin, isovalerylshikonin.	He, Qing, & Zhang, 2017
CN108409570A (06-03-2018)	*Arnebia euchroma*	Fast and efficient purification method comfrey acetyl shikonnin	Ethylacetate/petroleum ether	Reverse phase silica gel chromatography	Acetylshikonin	Jiang, Lin, & Zhai, 2018

## 5 Conclusion

Alkannin/shikonin and its derivatives possess a wide variety of pharmacological activities. These constituents are majorly investigated for their wound healing, antimicrobial and anticancer potential. In the last decade, various mechanisms of alkannin/shikonin and their derivatives are explored implicated in wide variety of diseases. The present study suggests the higher applicability of alkannin/shikonin and its derivatives are in the development of potent and safer wound healing and anticancer agents. Various analytical investigations are also discussed that will help the analysts for more efficient analysis of alkannin/shikonin and its derivatives from different sources. Brief patent summary is provided to highlight the future marketable potential of alkannin/shikonin and its derivatives. The appropriate knowledge of the pharmacological aspects of A/S and their derivatives will not only benefit the natural product researchers but also the pharmaceutical/formulation scientists in their future course of action. Further, the advanced and novel drug delivery systems could be used to mask the limitations of these derivatives including their low solubility and photo degradation. Despite having magnificent pharmacological potential, there is a dire need to collect remarkable data related to their toxicological and safety profile which can establish the clinical usage of these components.

## Declaration of Competing Interest

The authors declare that they have no known competing financial interests or personal relationships that could have appeared to influence the work reported in this paper.
